# Vibration Induces BAFF Overexpression and Aberrant O-Glycosylation of IgA1 in Cultured Human Tonsillar Mononuclear Cells in IgA Nephropathy

**DOI:** 10.1155/2016/9125960

**Published:** 2016-09-08

**Authors:** Muyao Ye, Youming Peng, Chan Liu, Wenzhe Yan, Xiaofei Peng, Liyu He, Hong Liu, Fuyou Liu

**Affiliations:** Nephrology Department, 2nd Xiangya Hospital, Central South University, Key Lab of Kidney Disease and Blood Purification, Changsha, Hunan 410011, China

## Abstract

*Objective*. To investigate the influence of* in vitro* vibratory stimulation of human tonsillar mononuclear cells (TMCs).* Methods*. Fourteen IgA nephropathy (IgAN) patients with chronic tonsillitis (CT) and 12 CT patients with no renal pathology were enrolled. Group A TMCs were collected after 24 hours of culture and used to determine baseline levels. TMCs in groups B, C, D, E, and F were exposed to vibratory stimulation (60 Hz) for 0 (as the control group), 1, 3, 5, and 10 minutes, respectively.* Results*. Baseline concentrations of B-cell-activation factor (BAFF) and IgA1, BAFF mRNA expression, and aberrant O-glycosylation IgA1 level were higher in the IgAN group as compared to that in the CT group, and all increased after vibratory stimulation. Baseline mRNA expressions of core *β*1,3-galactosyltransferase (C1GALT1) and core *β*1,3GalT-specific molecular chaperone (Cosmc) were lower in the IgAN group; the levels decreased further after vibratory stimulation.* Conclusion*. In patients with IgAN, vibratory stimulation of TMCs appears to induce IgA1 secretion through activation of BAFF release and to aberrant O-glycosylation IgA1 by suppressing C1GALT1 and Cosmc expression.* In vitro* vibratory stimulation of human TMCs mimics the vibratory simulation of palatine tonsils produced by vocal cords during phonation.

## 1. Introduction

 IgA nephropathy is one of the most common forms of primary glomerulonephritis in the world, including China [1]. It is characterized by the presence of immune deposits with dominant or codominant IgA in the glomerular mesangium of kidneys [2]. The prevalence of IgA nephropathy in Asia is 40% which is higher than that in Europe and North America (5%−10%) [3]. Aberrant glycosylation of IgA1 subclass is known to play an important role in the pathogenesis of IgA nephropathy [4–6]. In these patients, aberrantly glycosylated IgA1 molecules have been detected in kidneys, blood, urine, and lymphoid tissue in palatine tonsils [7, 8]. The pathogenesis of IgA nephropathy is closely related with the increasing of aberrant glycosylation IgA1 molecules, its aberrant deposits in mesangial area, and the abnormal metabolic disorders of IgA1 molecules [9]. Aberrant glycosylation results from deranged expression of enzymes core *β*1,3-galactosyltransferase (C1GALT1) and core *β*1,3GalT-specific molecular chaperone (Cosmc) [10, 11]. Patients with IgA nephropathy have been shown to have lower C1GALT1 activity in B cell lysates of blood [12]. The activity of C1GALT1 depends on the specific molecular chaperone Cosmc. Thus, reductions in C1GALT1 activity and Cosmc expression may both have a role to play in the aberrant glycosylation of IgA1 molecules [13]. As IgA1, C1GALT1 and Cosmc are all produced by B lymphocyte; B-cell-activation factor (BAFF) can enhance the expression of IgA molecules and their deposition in the kidneys and intestinal mucosa [14].

Infectious diseases such as acute tonsillitis are known to aggravate hematuria and proteinuria in IgAN patients. Early tonsillectomy is thought to reduce the risk of recurrence and promote long-term remission in patients with IgA nephropathy [15]. However, hematuria may aggravate during the week after tonsillectomy [16]. Studies conducted in Japan have documented aggravation of proteinuria, leukocytosis, and an increase in urinary sediment after stimulation of tonsils by ultra short waves for 10 minutes [17]. In addition, we observed an increased prevalence of IgAN among teachers, salespersons, and those involved in occupations that require increased vocalization.

Building on the above evidence, we investigated the influence of* in vitro* vibratory stimulation of human tonsillar mononuclear cells (TMCs) obtained from patients with IgA nephropathy (IgAN) and chronic tonsillitis (CT).

## 2. Materials and Methods

### 2.1. Subjects

Human tonsillar mononuclear cells (TMCs) were obtained from 14 (7 men and 7 women; mean age, 27.9 ± 8.7 years) patients with IgAN (biopsy confirmed) who were diagnosed with chronic tonsillitis (CT). A total of 12 (7 men and 5 women; mean age, 32.0 ± 12.1 years) patients with CT, but with no coexisting renal pathology were enrolled as controls. All IgAN patients had hematuria, while four had proteinuria. However, the renal function was normal in all subjects. All CT patients had normal urine and renal function tests (Table 1). Patients were enrolled at the Second Xiangya Hospital of Central South University from April 2013 to October 2014 and underwent tonsillectomy at the Department of Otolaryngology. Written informed consent was obtained from all participants. The study protocol was in compliance with the* Ethical Principles for Medical Research Involving Human Subjects* (World Medical Association Declaration of Helsinki, 2004). Due approval was obtained from the Clinical Ethics Committee at the Second Xiangya Hospital of Central South University.

### 2.2. Isolation and Culture of Tonsillar Mononuclear Cells (TMCs)

Surgically resected palatine tonsil specimens were washed thrice with sterile saline to remove blood cells on surface. The washed tissues were soaked for 15 mins in 20 mL PBS containing penicillin (500 *μ*g/mL) and streptomycin (500 *μ*g/mL). The tissue was ground into small pieces and filtered through a 200-mesh stainless steel sieve and the filtrate collected. An equal volume of lymphocyte separating medium was added to separate TMCs by density gradient centrifugation. Erythrocyte lysis buffer was added to decrease the excess red blood cells. After three irrigations with PBS, the cells were maintained in RPMI-1640 medium supplemented with 10% fetal calf serum (FCS), L-glutamine (2 mmol/L), penicillin (100 *μ*g/mL), and streptomycin (100 *μ*g/mL) in humidified 5% CO_2_ atmosphere at 37°C.

### 2.3. Mechanism of Vibratory Stimulation of TMCs

An electric vibratory massage device (60 Hz AC power) was used for vibratory stimulation of the cultured cells. A flat soft carbon particles package was kept on cleaned countertops and the culture bottle kept on the package to relieve the vibration intensity. The vibratory device was in direct contact of the surface of the culture bottle. This allowed for visualization of the vibrating cell suspension.

### 2.4. Controls and Experimental Design

Each sample was divided into six groups in separated culture bottles to avoid interaction of vibration. Group A specimens were collected after 24 hours of culture and used to determine baseline levels; group B specimens were cultured for another 72 hours, were not exposed to vibratory stimulation, and served as the control group; TMCs in groups C, D, E, and F were exposed to vibratory stimulation (60 Hz) for 1, 3, 5, and 10 minutes, respectively, and were cultured for another 72 hours under similar conditions.

### 2.5. Influence of Vibration on Cell Survival Ratio Measured by MTT Assay

Cell survival ratio was measured by the ratio of number of cells in each group to those in the base value group by MTT assay. MTT is a yellow dye used to distinguish living cells from dead cells. Living cells have enzymes which can change MTT into blue color formazan, while dead cells cannot. The optical density (OD) is measured at a wavelength of 490 nm by enzyme-linked immunosorbent assay. The OD value of each group is compared with that of base value group to indicate the survival ratio each group.

### 2.6. Enzyme-Linked Immunosorbent Assay

Culture supernatants were analyzed by specific ELISA for IgA1, aberrantly O-glycosylated IgA1, and BAFF. Mouse antihuman primary IgA1-specific antibody (Santa Cruz Biotechnology, USA) was fixed on the ELISA plate, followed by the sequential addition of human IgA1 protein standards (Calbiochem, USA), the samples, mouse biotin-labeled antihuman IgA-specific antibodies (Southern Biotech Associates, USA) for IgA1 while biotin-labeled lectin* Vicia* (Vector Laboratories Associates, USA) for aberrantly O-glycosylated IgA1, HRP-labeled Streptavidin (Beyotime Institute of Biotechnology, China), and TMB color liquid and stop solution (Beyotime Institute of Biotechnology, China). The optical density (OD) was measured at a wavelength of 450 nm by enzyme-linked immunosorbent assay to obtain concentrations of the supernatants. BAFF level was assessed by Human B-Cell-Activation Factor ELISA Kit (Cusabio, China), as per the manufacturer's recommendations.

### 2.7. Real-Time Quantitative PCR

Total RNA of TMCs was extracted by Trizol reagent (Invitrogen) and reverse transcription of total RNA was performed using the PrimeScript TM RT reagent kit with gDNA Eraser (TaKaRa, Japan) following the manufacturer's instructions. Together with GAPDH as the reference, BAFF, C1GALT1, and Cosmc were amplified using the primers as follows: BAFF: 5′-CCA CAG AAA GGG AGC AGT CAC-3′ (sense) and 5′-TGG GAG GAT GGA AAC ACA CT-3′ (antisense); C1GALT1: 5′-GAC CCT GAA GAA CCC ATT TAC TT-3′ (sense) and 5′-TAT CCT GCT CCT CCA CTC AT-3′; Cosmc: 5′-GAA GAT GCT GAT GGA AAA GAT g-3′ (sense) and 5′-CCT GGT TGG GGT GAT AAG TC-3′ (antisense). PCRs were performed in 20 *μ*L using the FastStart Universal SYBR Green Master with ROX (Roche, USA), and cDNA was adjusted with RNase-free dH2O to 1 *μ*g of RNA as template for reverse-transcription. We use ABI 7300 Real-time PCR System for the amplification reaction; it consists of 10 min of activation of FastStart Taq DNA Polymerase at 95°C, 40 cycles of 95°C/15 s, followed by 60°C/1 min for amplification and real-time analysis.

### 2.8. Statistical Analysis

All the data from ELISA and PCR are the results from triplicate experiments. Results are expressed as mean ± standard error of mean (SEM). Intergroup differences were assessed using Student's *t*-test. *P* ≤ 0.05 were considered statistically significant.

## 3. Results

### 3.1. Influence of Vibration on Cell Survival Ratio

**Figure 1:**
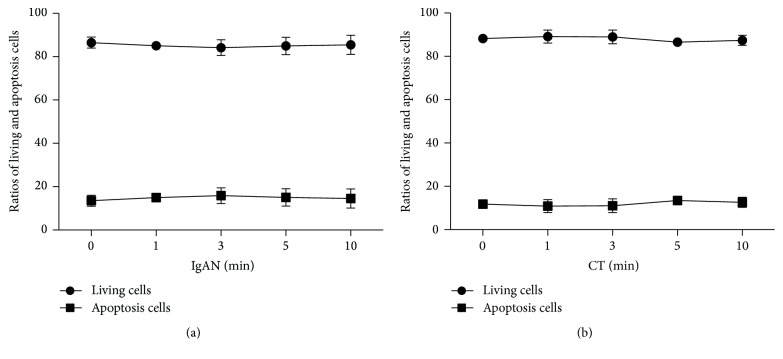
Cell survival ratio in the IgAN (a) and CT groups (b) after exposure to vibratory stimulus for varying durations of time. Vibration did not induce significant changes in cell survival ratio (*P* > 0.05). Data expressed as mean ± SEM. IgAN, Immunoglobulin A nephropathy; CT, chronic tonsillitis; SEM, standard error of the mean.

When exposed to vibrations for >10 minutes, some culture bottles developed minute cracks which may have contaminated the cell cultures. To avoid this in subsequent experiments, we limited the vibration time to 10 minutes. The results showed that vibratory stimulus did not affect cell survival ratio. Exposure to vibration for different time durations resulted in a minor effect on TMCs survival ratio in both the groups (Figure 1). However, the difference was not statistically significant (*P* > 0.05).

### 3.2. Influence of Vibration on IgA1 and Aberrantly O-Glycosylated IgA1 Release

**Figure 2:**
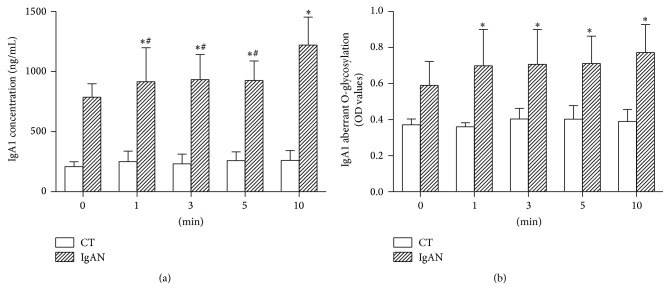
Concentration of IgA1 (a) and aberrant O-glycosylation IgA1 level (b) in the supernatant of cells subjected to different time periods of vibration. Data are expressed as mean ± SEM. *∗* indicates significant difference from control (0 min) of IgAN group (*P* < 0.05). # in (a) indicates significant difference from the IgAN group exposed to vibrations for 10 min (*P* < 0.05). IgAN, Immunoglobulin A nephropathy; SEM, standard error of the mean.

Baseline IgA1 concentration in the IgAN group was significantly higher than that in the CT group (844.0 ± 112.9 ng/mL versus 207.2 ± 61.5 ng/mL, *P* < 0.001). Exposure to vibration caused an increase in IgA1 concentration in the IgAN group. The IgA1 concentration after 10 mins of vibratory stimulus was higher as compared to those measured after 1, 3, or 5 mins of exposure (Figure 2(a)). At baseline, the aberrant O-glycosylation IgA1 level in the IgAN group was significantly higher than that in the CT group (OD value: 0.59 ± 0.13 versus 0.37 ± 0.03, *P* < 0.001). Exposure to vibration caused an increase in the aberrant O-glycosylation IgA1 levels in the IgAN group, with no significant difference for different time periods (Figure 2(b)).

### 3.3. Influence of Vibration on BAFF mRNA Expression and BAFF Release

**Figure 3:**
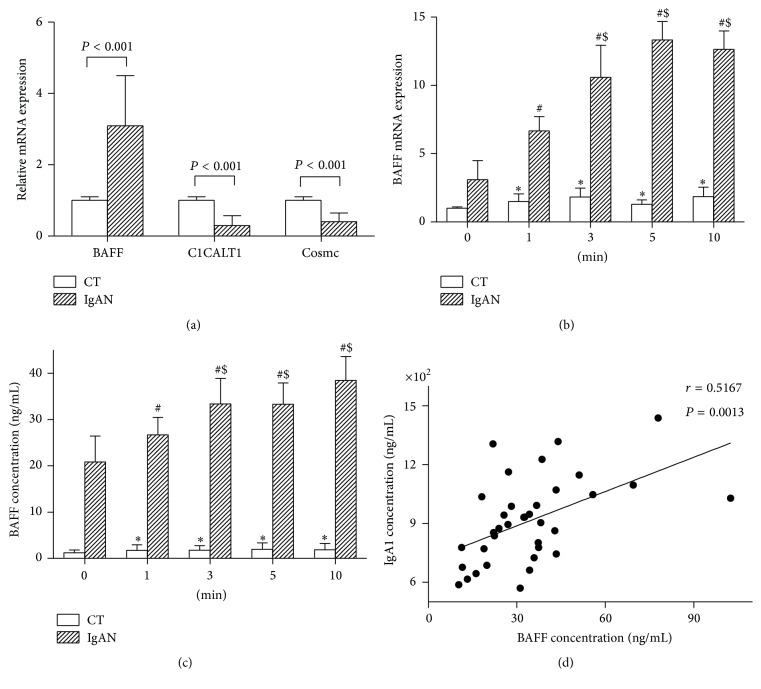
BAFF mRNA expression (b) and BAFF concentration (c) in TMC cells subjected to different time periods of vibration. (a) is the baseline values of BAFF, C1GALT1, and Cosmc in the two groups. Data are expressed as mean ± SEM. *∗* indicates significant difference from control (0 min) of CT group (*P* < 0.05). # indicates significant difference from control (0 min) of IgAN group (*P* < 0.05). $ indicates significant difference from the subgroup exposed to vibratory stimulation for 1 min of the IgAN group (*P* < 0.05). (d) shows positive correlation between BAFF and IgA1 concentration (*r* = 0.5167, *P* = 0.0013). B-cell-activation factor (BAFF); TMC, tonsillar mononuclear cells; C1GALT1, core *β*1,3-galactosyltransferase; Cosmc, core *β*1,3GalT-specific molecular chaperone; IgAN, Immunoglobulin A nephropathy; CT, chronic tonsillitis; SEM, standard error of the mean.

Baseline BAFF mRNA expression (Figure 3(a)) and BAFF concentration were significantly higher in the IgAN group as compared to that in the CT group (13.25 ± 5.09 ng/mL versus 1.47 ± 0.55 ng/mL, *P* < 0.001). Further, exposure to vibratory stimulation caused an increase in both BAFF mRNA expression and BAFF concentration in the IgAN group (Figures 3(b) and 3(c)). The gene expressions after 3, 5, or 10 minutes of vibratory stimulation were higher than that vibrated with 1 minute in the IgAN group. In the CT group, vibrations caused an increase in BAFF expression and release; however, there were no differences between different time periods. Although vibrations caused an increase in BAFF in both the groups, the increasing degrees of BAFF mRNA expression were found to be similar when vibrated for 1 minute. When duration of exposure to vibrations exceeded 1 minute, the extent of increase in the IgAN group was higher than that in the CT group (Table 2). Pearson correlation analysis showed a positive correlation between BAFF and IgA1 concentration in the IgAN group (Figure 3(d)).

### 3.4. Influence of Vibration on C1GALT1 and Cosmc mRNA Expression

**Figure 4:**
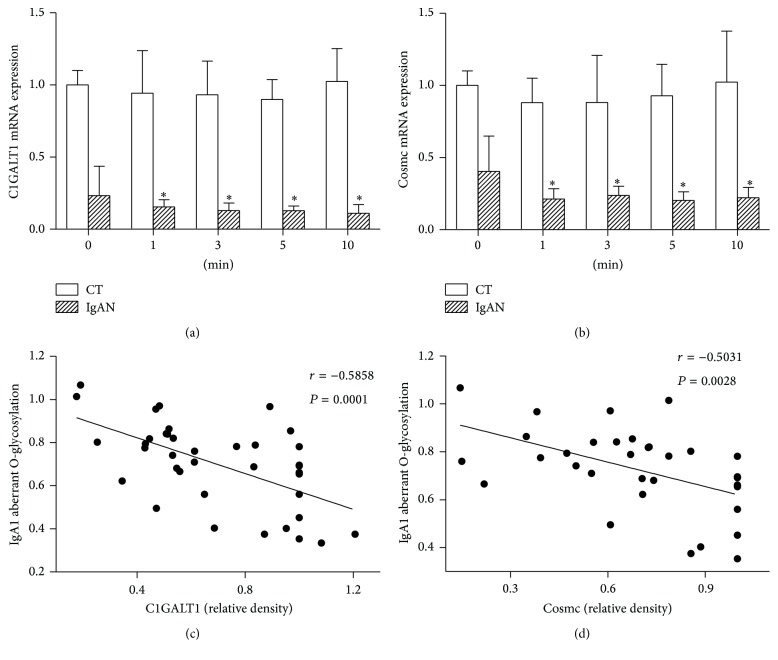
The gene expression of C1GALT1 (a) and Cosmc (b) mRNA of TMCs subjected to different time periods of vibration. Data are mean ± SEM. The symbol *∗* indicates significant difference from control (0 min) of IgAN group (*P* < 0.05). (c) and (d) show negative correlation between aberrant O-glycosylation IgA1 level and C1GALT1 (*r* = −0.5858, *P* = 0.0001) and aberrant O-glycosylation IgA1 level and Cosmc (*r* = −0.5031, *P* = 0.0028). TMC, tonsillar mononuclear cells; C1GALT1, core *β*1,3-galactosyltransferase; Cosmc, core *β*1,3GalT-specific molecular chaperone; IgAN, Immunoglobulin A nephropathy; SEM, standard error of the mean.

The mRNA expression of C1GALT1 and Cosmc at baseline was significantly lower in the IgAN group (Figure 3(a)). Vibration caused a decrease in C1GALT1 and Cosmc expression in the IgAN group. However, there were no significant intergroup differences between different time durations of exposure. No corresponding effect was observed in the CT group (Figures 4(a) and 4(b)). Pearson correlation analysis showed a negative correlation between aberrant O-glycosylation IgA1 level and C1GALT1 and Cosmc mRNA expression (Figures 4(c) and 4(d)).

## 4. Discussion

Due to anatomical proximity, vocal cord vibration is likely to physically stimulate palatine tonsils during phonation. The experimental setting allowed for* in vitro* exposure of cells to vibratory stimuli. However, the easy mechanism was limited for the frequency and amplitude of the vibratory stimulus, and we set it to frequency of 60 Hz. The vibratory stimulus was intended to mimic the natural vibrations generated from vocal chords during the act of a normal conversation in real life.

*In vitro* vibratory stimulation of TMCs obtained from IgAN patients caused increase in IgA1 concentration and aberrant O-glycosylation IgA1 and BAFF mRNA expression and concentration, while it caused a decrease in C1GALT1 and Cosmc mRNA expression. In the CT patients, this vibration only increased BAFF mRNA expression and its concentration. These results suggest that TMCs vibration could trigger aberrant tonsillar mucosal immune responses. The normal mucosal immune responses in CT patients are via production of IgG by palatine tonsils. In IgAN patients, tonsils are known to produce more IgA [18]. The imbalance of both mucosal immunity and systematic immunity can lead to the progression of IgAN. Since IgA nephropathy is considered as an autoimmune disease related to the mucosal immune responses, numerous studies have explored whether tonsillectomy can effectively improve the prognosis of patients with IgA nephropathy. A meta-analysis of studies from Asia indicated a significantly higher clinical remission rate at 10 years, in patients who underwent palatine tonsillectomy [19]. Another study showed significantly lower serum IgA levels in tonsillectomized patients as compared to those in nontonsillectomized subjects [20]. Palatine tonsils are located at the entrance of the airway, with the mouth serving as a resonant cavity. The continuous high intensity vocal vibrations can cause physical stimulation of the entire airway as well as of the palatine tonsils. Studies have shown that mechanical stress from vibrations produced during snoring may induce inflammation of airway tissues [21]. Further, obstructive sleep apnea syndrome (OSAS) is closely associated with asthma [22]. In clinical practice, some patients engaged in occupations that require speaking loudly for long period of times, such as teachers and salespersons, often suffer from frequent episodes of hematuria, which tends to be of a greater severity than that in other patients. Therefore, we speculate that sustained high intensity of vocal cord vibration may influence the palatine tonsils to some extent, causing immune abnormalities of varying degrees, thereby giving rise to the development of IgA nephropathy. Although speaking produces a complex sound extended up to high frequencies, spectrum analysis has shown a high-amplitude vibration at low frequencies (50–100 Hz) [23]. Given that the frequency of the available vibration setting is 60 Hz, we selected a frequency of 60 Hz to study the response of TMCs to vibration.

Among the different types of cells, we focused on lymphocytes to assess the potential injurious effects of vibration because of the important role of B lymphocytes in pathogenesis of IgA nephropathy. B lymphocytes produce IgA1 and can be stimulated by B-cell-activation factor (BAFF). BAFF is mainly produced by peripheral blood mononuclear cells, including neutrophils, monocytes, macrophages, and dendritic cells [24]. BAFF overexpression may cause significant increase in mature B cells and affect T cells, and high levels of BAFF were associated with characteristics akin to that of autoimmune disorders, such as high concentrations of circulating immune complexes and immunoglobulin deposition in kidney [25]. Serum BAFF levels were significantly higher in patients with IgA nephropathy [26]. As abnormal IgA1 is secreted by B lymphocytes, BAFF appears to be closely related with IgA nephropathy [27]. BAFF-specific antagonist was shown to inhibit the activity of BAFF and may thus be a new therapeutic target for some autoimmune diseases [28].

In this experiment, IgA1 concentration, BAFF concentration, and BAFF mRNA expression at baseline were significantly higher in the IgAN group, as compared to that in the CT group. After exposure to vibrations for different durations of time, IgA1 concentration increased in the IgAN group, while BAFF concentration and BAFF mRNA expression increased in both the groups. On vibratory stimulation for 3, 5, or 10 minutes, both BAFF concentration and BAFF mRNA expression were higher than that on vibratory stimulation for 1 minute. BAFF mRNA increase in the IgAN group was higher than that in the CT group, except for vibratory stimulation for 1 minute. Pearson analysis showed positive correlation between BAFF and IgA1 concentration in the IgAN group, which indicates that BAFF may be one of the causative factors in pathogenesis of IgA nephropathy. In the CT group, BAFF increased while IgA1 concentration remained unchanged. It shows that BAFF may be more sensitive to vibratory stimulation, but the changes in the CT group were not enough to induce secretion of immunoglobulins, such as IgA1. With prolonged vibratory stimulation, BAFF expression did not increase in the CT group, while there was significant increase in the IgAN group. After vibration, we found aberrant O-glycosylation of IgA1 increased in the IgAN group, alongside a decrease in C1GALT1 and Cosmc expression; no changes were observed in the CT group.

## 5. Conclusion

Our study provides experimental evidence of aberrant mucosal immune response induced by vibratory stimulation of cultured TMCs. The findings are consistent with the hypothesis that vibrations produced during speaking could be an important contributor to the aberrant tonsillar mucosal immune responses observed in patients with IgA nephropathy who by virtue of their vocation require a lot of speaking. The simple experimental setting developed in this work can be used to study the effect of vibration on other cell types in the tonsillar and peritonsillar tissues that may be affected by vibrations induced by speaking (proinflammatory response, macrophages, etc.). Such experiments may help unravel the cellular mechanism of tonsillar mucosal immune dysfunction associated with excessive phonation in patients with IgA nephropathy. A better understanding may help develop innovative antiautoimmune therapies in these patients.

## Figures and Tables

**Table 1:** Patients' information of IgAN and CT group (mean ± SEM).

	IgAN	CT	*P*
Total	14	12	NT
Age	27.9 ± 8.7	32.0 ± 12.1	NS
Male/female	7/7	7/5	NS
Systolic pressure (mmHg)	113.8 ± 10.7	11.4 ± 17.2	NS
Diastolic pressure (mmHg)	74.3 ± 7.5	75.2 ± 9.7	NS
BUN (mmol/L)	5.3 ± 1.3	4.9 ± 1.4	NS
SCR (*μ*mol/L)	84.0 ± 23.4	58.1 ± 14.9	0.007
UA (*μ*mol/L)	351.8 ± 99.4	324.3 ± 94.3	NS
Alb (g/L)	36.6 ± 3.3	41.7 ± 2.4	0.002
Hb (g/L)	124.3 ± 17.2	140.9 ± 20.4	NS
Urinary protein (g/L)	1.8 ± 2.2	—	NT
Hematuria (Ery/*μ*L)	165 ± 135.8	—	NT

—: negative; NS: no significance; NT: not tested.

**Table 2:** Effect of vibratory stimulation on increasing degrees of BAFF mRNA expression in human TMCs.

	Duration of exposure			
	1 min	3 min	5 min	10 min
IgAN	2.16 ± 0.75	3.43 ± 1.67	4.31 ± 0.96	4.09 ± 0.96
CT	1.50 ± 0.56	1.83 ± 0.65	1.30 ± 0.32	1.85 ± 0.69
*P* value	0.116	0.021	0.002	<0.001

BAFF, B-cell-activation factor; TMCs, tonsillar mononuclear cells; IgAN, IgA nephropathy; CT, chronic tonsillitis.
